# Phylogeographic Refinement and Large Scale Genotyping of Human Y Chromosome Haplogroup E Provide New Insights into the Dispersal of Early Pastoralists in the African Continent

**DOI:** 10.1093/gbe/evv118

**Published:** 2015-06-24

**Authors:** Beniamino Trombetta, Eugenia D’Atanasio, Andrea Massaia, Marco Ippoliti, Alfredo Coppa, Francesca Candilio, Valentina Coia, Gianluca Russo, Jean-Michel Dugoujon, Pedro Moral, Nejat Akar, Daniele Sellitto, Guido Valesini, Andrea Novelletto, Rosaria Scozzari, Fulvio Cruciani

**Affiliations:** ^1^Dipartimento di Biologia e Biotecnologie “C. Darwin,” Sapienza Università di Roma, Italy; ^2^Dipartimento di Biologia Ambientale, Sapienza Università di Roma, Italy; ^3^Accademia Europea di Bolzano (EURAC), Istituto per le Mummie e l'Iceman, Bolzano, Italy; ^4^Dipartimento di Sanità Pubblica e Malattie Infettive, Sapienza Università di Roma, Italy; ^5^Laboratoire d’Anthropologie Moléculaire et Imagerie de Synthèse, UMR 5288, Centre National de la Recherche Scientifique (CNRS), Université Toulouse-3–Paul-Sabatier, Toulouse, France; ^6^Department of Animal Biology-Anthropology, Biodiversity Research Institute, University of Barcelona, Spain; ^7^Pediatrics Department, TOBB-Economy and Technology University Hospital, Ankara, Turkey; ^8^Istituto di Biologia e Patologia Molecolari, CNR, Rome Italy; ^9^Dipartimento di Medicina Interna e Specialità Mediche, Sapienza Università di Roma, Italy; ^10^Dipartimento di Biologia, Università di Roma “Tor Vergata,” Italy; ^11^ Present address: The Wellcome Trust Sanger Institute, Hinxton, Cambridgeshire, United Kingdom

**Keywords:** human Y chromosome, African prehistory, MSY phylogeny, dispersal of early pastoralists, next generation sequencing, SNP-based dating

## Abstract

Haplogroup E, defined by mutation M40, is the most common human Y chromosome clade within Africa. To increase the level of resolution of haplogroup E, we disclosed the phylogenetic relationships among 729 mutations found in 33 haplogroup DE Y-chromosomes sequenced at high coverage in previous studies. Additionally, we dissected the E-M35 subclade by genotyping 62 informative markers in 5,222 samples from 118 worldwide populations. The phylogeny of haplogroup E showed novel features compared with the previous topology, including a new basal dichotomy. Within haplogroup E-M35, we resolved all the previously known polytomies and assigned all the E-M35* chromosomes to five new different clades, all belonging to a newly identified subhaplogroup (E-V1515), which accounts for almost half of the E-M35 chromosomes from the Horn of Africa. Moreover, using a Bayesian phylogeographic analysis and a single nucleotide polymorphism-based approach we localized and dated the origin of this new lineage in the northern part of the Horn, about 12 ka. Time frames, phylogenetic structuring, and sociogeographic distribution of E-V1515 and its subclades are consistent with a multistep demic spread of pastoralism within north-eastern Africa and its subsequent diffusion to subequatorial areas. In addition, our results increase the discriminative power of the E-M35 haplogroup for use in forensic genetics through the identification of new ancestry-informative markers.

## Introduction

The Male-Specific portion of the human Y chromosome (MSY) is an invaluable tool to investigate many issues about population history (Jobling and Tyler-Smith 2003) and forensic genetics (Jobling 2001; Kayser et al. 2007). Both the lack of meiotic recombination and the uniparental inheritance imply that the MSY differentiation may only be generated by the sequential accumulation of new mutations along radiating male-borne lineages (Underhill and Kivisild 2007). This process creates monophyletic and evolutionary stable entities known as “haplogroups,” defined by biallelic markers (usually single nucleotide polymorphisms [SNPs]) with a low mutation rate (Xue et al. 2009), which can be arranged in an unambiguous maximum parsimony (MP) phylogenetic tree (Underhill et al. 2000; Karafet et al. 2008; van Oven et al. 2014).

Taking into account the phylogenetic information and the ethnogeographic distribution of haplogroups (i.e., the phylogeographic approach), it is possible to understand and to date some demographic processes behind the dispersal of human populations (for a review, see Chiaroni et al. 2009). Furthermore, by analyzing the worldwide ethnogeographic distribution of different biallelic markers, it is often possible to find one (or more) specific haplogroup confined to restricted geographic areas, allowing the identification of the likely place of origin of an individual carrier (Mizuno et al. 2010; Cruciani et al. 2011; Larmuseau et al. 2011, 2012; Geppert et al. 2015).

In recent years, the advent of Next Generation Sequencing (NGS) led to the discovery of thousands of new polymorphisms used to improve the Y chromosome phylogenetic tree. In addition, the possibility of revealing a high number of stable polymorphisms has led to the reevaluation of SNPs as an optimal tool for age estimation of the tree nodes (Francalacci et al. 2013; Poznik et al. 2013; Wei, Ayub, Chen, et al. 2013; Scozzari et al. 2014; Lippold et al. 2014; Hallast et al. 2015). Major changes within the Y phylogeny, obtained by NGS analyses, involved both the resolution of some deep multifurcations (Francalacci et al. 2013; Poznik et al. 2013) and the identification of haplogroups marking recent demographic expansions (Wei et al. 2013; Yan et al. 2014; Hallast et al. 2015).

Despite the notable progresses in SNPs discovery, a deep-sequencing-based analysis of the internal diversity of some specific Y haplogroups is still lacking. Probably, this is because a comprehensive description of the internal diversity of a specific haplogroup requires a targeted sampling of widely divergent chromosomes (Scozzari et al. 2014) rather than a population-based sampling design (Francalacci et al. 2013; Poznik et al. 2013; Wei et al. 2013; Lippold et al. 2014; Hallast et al. 2015).

In this study, we present an updated phylogenetic structure for haplogroup E, which is the most represented MSY lineage in Africa (Cruciani et al. 2002), focusing on the E-M35 clade. This haplogroup received considerable attention in the literature because it has a broad geographic distribution, being present at high frequencies in a wide area stretching from northern and eastern Africa to Europe and western Asia. Moreover, the lineages sharing the M35 mutation have been linked to a wide range of human movements and a multitude of theories have been proposed about their time and place of origin (Arredi et al. 2004; Semino et al. 2004; Cruciani et al. 2006, 2007; Adams et al. 2008; Henn et al. 2008; Battaglia et al. 2009; Lancaster 2009; Trombetta et al. 2011; Bučková et al. 2013; Gebremeskel and Ibrahim 2014). Although several studies have progressively improved the phyletic resolution of this clade (Cruciani et al. 2004, 2006, 2007; Trombetta et al. 2011), it continued to display different polytomies and phylogenetic uncertainties (Trombetta et al. 2011; Lippold et al. 2014; van Oven et al. 2014), with a relatively high number of chromosomes still belonging to the paraphyletic E-M35* paragroup (Cruciani et al. 2004; Trombetta et al. 2011).

In this study, we analyzed 33 high coverage sequences from different data sets (Drmanac et al. 2010; Scozzari et al. 2014) to reconstruct a high confidence haplogroup E phylogeny based on 729 mutations. We used 62 variants to identify and genotype 1,141 E-M35 chromosomes selected from a wider pool of 5,222 males from 118 worldwide populations.

By this analysis, we provided a high-resolution branching of haplogroup E-M35, resolving its previous multifurcations. Furthermore, we assigned all E-M35* chromosomes to new specific terminal branches and identified geographically restricted E-M35 subhaplogroups, thus increasing the discriminative power of the haplogroup for use in human evolution and forensics. Finally, we identified a new monophyletic clade, which accounts for about 40% of the E-M35 chromosomes from the Horn of Africa. SNP-based dating and phylogenetic structuring of this haplogroup highlight eastern Africa as a major center for the demic diffusion of early pastoralists within the continent.

## Materials and Methods

### Sample

Samples were obtained from peripheral blood, cultured cells or buccal swab and DNA was extracted using appropriate procedures.

The “sequencing” set consisted of 33 male subjects coming from three different data sets: 1) 18 haplogroup E subjects sequenced in the same experiment of, but not described in detail in Scozzari et al. (2014); 2) three haplogroup E males yet unpublished; and 3) 12 haplogroup DE unrelated samples from the diversity panel of the Complete Genomics company (Drmanac et al. 2010). The haplogroup affiliation and the geographic provenance of the sequenced subjects are described in supplementary table S1, Supplementary Material online.

The “genotyping” set consisted of 5,222 males from 118 populations worldwide, which were analyzed for specific informative markers.

### NGS Sequencing, Data Quality Control, and Filtering

For the 21 subjects sequenced in this study and Scozzari et al. (2014), DNA quality control, library preparation, sequencing and data filtering were as reported therein. Overall, 1,495,512 bases of the target region, subdivided into 5,274 baited fragments, were resequenced. Genotyping results (VCF files) for the genomes of the 12 subjects of data set (3) were downloaded from the FTP site of the Complete Genomics company (ftp://ftp2.completegenomics.com, last accessed June 30, 2015). For each VCF file, we extracted single nucleotide substitutions falling within the same 1,495,512 bases mentioned above. For each sample from Complete genomics, low quality mutations (FT = VqLow) and/or mutations with allelic depth ≤ 2 were discarded.

### Phylogenetic Analysis and Dating

Phylogenetic analysis among variants identified by sequencing was independently performed using MEGA (Tamura et al. 2011) and NETWORK (Bandelt et al. 1999). The .out file produced by this latter program was manipulated in a spreadsheet to directly obtain the assignment of mutations to each of the tree branches.

We applied two independent methods for dating the tree nodes. The first is based on the rho statistic, that is, the average number of differing sites between a set of sequences and a specified common ancestor (Forster et al. 1996). This statistic is linearly related to time and mutation rate (rho = μ × t) (Jobling et al. 2004), assuming constancy of the rate across the tree branches. The statistic and associated confidence interval were computed with the program Network (Bandelt et al. 1999). The second method is based on a Bayesian estimation of node ages, through BEAST. An initial run was used to obtain a seeding tree to be used in all subsequent runs. In all runs, we used a GTR (general time reversible) model for nucleotide substitutions under a strict clock. An expansion model was used for the population size, to appropriately account for the faster recent growth, and rather flat priors were used, that is, lognormal[10,3] for the current population size, exp[0.2] for the ancestral/current population size ratio, and uniform[0, 0.00133] for the growth rate/year in the expansion phase. We used runs of 10 million steps each, sampled every 10,000 steps. The initial 20% of each run was discarded as burn-in and the outputs analyzed with Tracer and Tree Annotator. Both for rho and BEAST estimates, we used a fossil-based substitution rate estimate of 0.716 × 10^−^^9^/site/year, which is specific for the 1.5-Mb MSY region here analyzed (Trombetta B, unpublished data) obtained by calibration with two archeologically dated specimens (Fu et al. 2014; Lazaridis et al. 2014).

### Genotyping

Overall, 5,222 samples were analyzed for markers M40 and M215, which define haplogroup E and E-M215, respectively. Samples carrying the derived allele for both markers were further analyzed for the M35 mutation. M35-derived chromosomes were hierarchically genotyped for 59 additional mutations whereas M215*(×M35) chromosomes were analyzed for the V16 mutation (supplementary table S2, Supplementary Material online). Among samples belonging to paragroup E-M40*(×M215), we found three samples belonging to the deep paragroup E-M40*(×M75, P147) (Cruciani F, Trombetta B, unpublished data), which were further genotyped for 13 mutations phylogenetically equivalent to M40 and for four additional markers (V44, V1844, V1865, and V3725) defining basal branches of haplogroup E (supplementary table S3, Supplementary Material online). The genotyping was performed by either Sanger Sequencing, polymerase chain reaction restriction fragment length polymorphism (PCR-RFLP) or DHPLC. We designed PCR and sequence primers (both available on request) on the basis of the Y-chromosome sequence reported in the February 2009 assembly of the UCSC Genome Browser (http://genome.ucsc.edu/, last accessed June 30, 2015) using Primer3 software (http://primer3.ut.ee, last accessed June 30, 2015). Sequencing, RFLP, and DHPLC templates were obtained through PCR in a 50-µl reaction containing 20 ng of genomic DNA, 200 mM each deoxyribonucleotide, 2.5 mM MgCl_2_, 1 unit of Taq polymerase, and 10 pmol of each primer. A touch-down PCR program was used with an annealing temperature that decreased from 62 to 55 °C for 14 cycles, followed by 30 cycles with an annealing temperature of 55 °C. Following DNA amplification, PCR products of markers analyzed by sequencing were purified using the QIAquick PCR purification kit (Qiagen, Hilden, Germany). Cycle sequencing was performed using the BigDye Terminator Cycle Sequencing Kit with Amplitaq DNA polymerase (Applied Biosystems, Foster City, CA) and an internal or PCR primer. Cycle sequencing products were purified by ethanol precipitation and run on an ABI Prism 3730XL DNA sequencer (Applied Biosystems). Chromatograms were aligned and analyzed for mutations using Sequencher 4.8 (Gene Codes Corporation, Ann Arbor, MI).

### Phylogeographic Analysis and Frequency Maps

In order to make inferences on the most likely locations for ancestors corresponding to nodes in the tree, terminal branches of the tree were assigned to six geographic macroregions, that is, Central-Western Africa, Southern Africa, Eastern Africa, Northern Africa, Europe, and rest of the world (comprising Near East and other continents). In most cases, the assignment to specific macroregion/s was based on the observed geographic distribution in our sample set (supplemented with data from literature for previously known branches). For some new E-M2 and E-V13 leaves, in absence of frequency distribution information, the macroregion assignment was based on the origin of the single sequenced sample. A discrete phylogeographic model was examined by Bayesian search implemented in the program RASP (Yu et al. 2010). This analysis was applied to the MP tree, allowing ancestral ranges to include no more than one of the six geographic macroregions. Inferences on ancestral locations for each node were represented as a pie chart overlaid on the tree.

Frequency maps for E-V1515 haplogroup and subhaplogroups were depicted on a grid of 50 × 45 lines using the Kriging procedure (Cressie 1991) through the use of the program Surfer 6.0 (Golden Software, Inc., Golden, CO). Data used for the frequency maps are from this study, supplemented with data reported by Gomes et al. (2010) and by Henn et al. (2008) for Uganda (one population) and Tanzania (seven populations), respectively. In both these studies, markers M35 and M293 have been analyzed. For the population from Uganda, we inferred the frequency of E-V1515 subhaplogroups (among chromosomes classified as E-M35* × M293) through Y-STR (short tandem repeats) comparisons (supplementary table S4, Supplementary Material online). For the seven populations from Tanzania, we approximated to zero the frequency of E-V1515 subhaplogroups other than E-M293, based on a paragroup E-M35*(×M293) reported frequency of 0% (four populations) and 4–5% (three populations).

## Results

### Haplogroup E Phylogeny and Dating

We disclosed the phylogenetic relationships among 729 mutations (supplementary table S5, Supplementary Material online) found in 1.5 Mb of 33 haplogroup DE Y-chromosomes (supplementary table S1, Supplementary Material online) that were sequenced at high-coverage (about 50×) in the present or previous studies (Drmanac et al. 2010; Scozzari et al. 2014). Although most of the 729 positions were already found to be variable (Drmanac et al. 2010; Wei et al. 2013; Scozzari et al. 2014), the phylogenetic relationships among those belonging to haplogroup E-M35 remained largely unexplored, making these polymorphisms potentially informative SNPs for forensic research, caseworks, and databases.

**Fig. 1.**
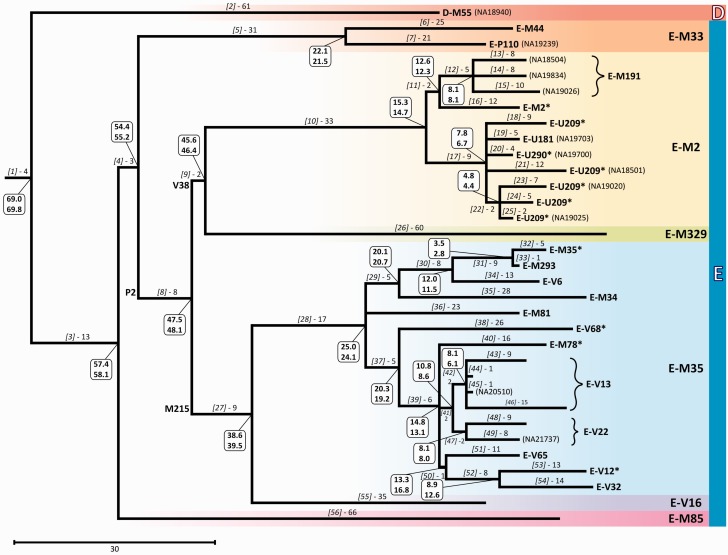
— MP tree of haplogroup E obtained with 729 variable positions. Above each branch are reported the haplogroup nomenclature used in the main text (in square brackets) and the number of mutational events defining each branch. Few internal key mutations are indicated as an aid to the reader. Dating estimates are reported in boxes near each node (the values on top and on bottom were obtained with BEAST and rho method, respectively). At the tip of each terminal branch, the haplogroup affiliation of the sequenced sample is reported, using a mutation-based nomenclature according to Trombetta et al. (2011). The ID of the samples from Complete Genomics is also indicated (in parentheses). Colored belts indicate major lineages.

We used the 729 haplogroup E DNA variants to reconstruct an MP patrilineal tree using two independent methods that produced trees with identical topology, and indicated no recurrent or double-hit mutations within the phylogeny. The MP tree is shown in figure 1.

Haplogroup E is here defined by 13 mutations (branch 3) (supplementary table S5, Supplementary Material online) and the phylogenetic relationships among the major internal branches of this clade (fig. 1) are consistent with the known topology (Trombetta et al. 2011; van Oven et al. 2014). However, new phylogenetic features emerged both within the E-M35 lineage (branch 28 in fig. 1) and among the basal branches of haplogroup E, after genotyping additional DNA samples (see subsequent sections).

In addition to carrying new information about phylogeny, the polymorphisms here analyzed are informative about the timing of lineage divergence. For each node in the tree, we obtained dating estimates using both the rho method (Forster et al. 1996) and a Bayesian approach implemented in BEAST (Drummond and Rambaut 2007) (fig. 1), using a nucleotide substitution rate of 0.716 × 10^−^^9^/site/year specific for the MSY regions here analyzed. Dating estimates are reported in supplementary table S6, Supplementary Material online. As the two methods produced highly concordant values (*r* = 0.997; supplementary fig. S1, Supplementary Material online), hereafter we only refer to the results obtained using BEAST. The TMRCA (time to the most recent common ancestor) for all haplogroup E chromosomes here analyzed is 57.4 ka (95% CI: 50.0–68.0 ka), older than the estimates of about 37 ka reported by Semino et al. (2004) and Hallast et al. (2015) using an STR- and SNP-based dating, respectively. These inconsistencies in TMRCA estimates are probably due to Y-STR mutation count saturation (Wei, Ayub, Xue, et al. 2013) in the first case (Semino et al. 2004) and to the use of a different SNP mutation rate (1.0 × 10^−^^9^/mutations/nucleotide/year) in the second case (Hallast et al. 2015). In the subsequent 20 ky, four major dichotomies were observed: 1) The split between E-M33 and E-P2 subhaplogroups, dated at 54.4 ka (95% CI: 47.4–64.4 ka); 2) the node separating E-V38 and E-M215 branches (47.5 ka; 95% CI: 41.3–56.8 ka); and 3) two unexpectedly old (45.6 and 38.6 ka; 95% CI: 38.4–53.7 and 31.4–45.9 ka, respectively) nodes which separate two common and widespread African haplogroups (E-M2 and E-M35) from two rare eastern African lineages (E-M329 and E-V16), respectively. Another striking aspect of our dating is the previously unappreciated large difference in the age between haplogroup E-M215 (38.6 ka; 95% CI: 31.4–45.9 ka) and its subhaplogroup E-M35 (25.0 ka; 95% CI: 20.0–30.0 ka). Within the E-V68 subclade, the M78 mutation arose in a time window between 20.3 ka (95% CI: 16.2–25.4 ka) and 14.8 ka (95% CI: 11.6–18.5 ka), namely the TMRCA for E-V68 and E-M78, respectively. The TMRCA of E-V13 chromosomes (8.1 ka; 95% CI: 5.6–10.8 ka) is consistent with a previous hypothesis about a post-Neolithic expansion of this haplogroup in Europe (Cruciani et al. 2004, 2007). Finally, the young TMRCA (3.5 ka; 95% CI: 1.7–5.9 ka) for the node separating the sequenced (former) E-M35* and E-M293 samples suggests a TMRCA for the M293 variant more recent than previously hypothesized using an STR-based dating (11.4 ka; Henn et al. [2008]).

### Refinement of E-M35 Internal Phylogeny through the Genotyping of Additional Samples

To refine the phylogeny of subhaplogroup E-M35, and to obtain new insights into its geographic distribution, a total of 62 mutations (supplementary table S2, Supplementary Material online) were hierarchically genotyped in 5,222 samples belonging to 118 populations. This screening included mutations obtained by NGS of 1.5 Mb of the MSY, plus others known to lie outside the sequenced regions, several of which poorly characterized at the population level. We found a total of 1,147 E-M215 Y chromosomes, most of which (1,141) belonging to the major E-M35 subclade (supplementary table S7, Supplementary Material online).

**Fig. 2.**
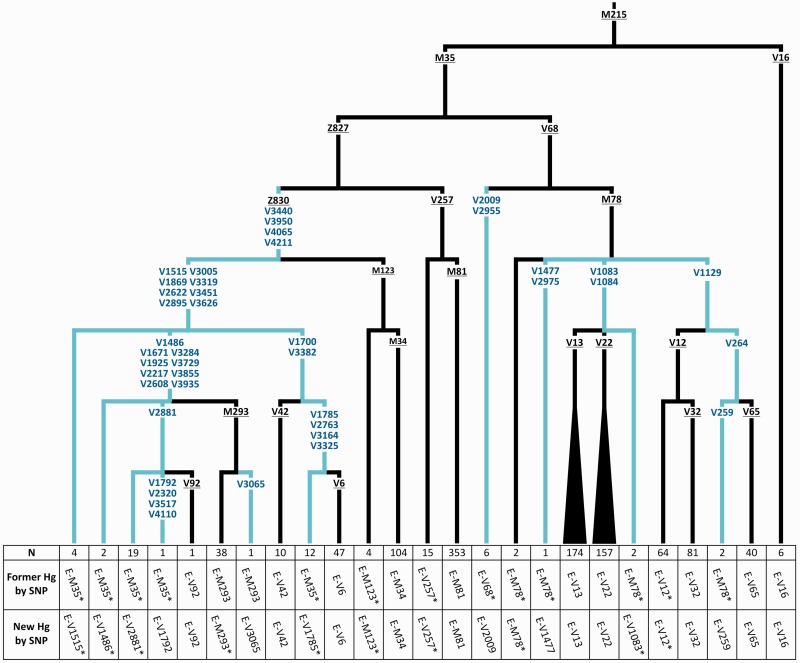
—Molecular dissection of haplogroup E-M35. All the 62 mutations shown have been genotyped. New mutations and branches are in blue. Previously known mutations are underlined. At the bottom of each branch are reported: The number of samples carrying the haplogroup, the former mutation-based nomenclature (according to Trombetta et al. [2011]), and the new mutation-based nomenclature. Chromosomes E-V1785* are ancestral for V1472, V1617, V2793, V2684, V2727, V2802, and V2927 (not shown).

The incorporation of the information obtained from this analysis into the previously reported E-M35 tree (Karafet et al. 2008; Trombetta et al. 2011) produced an extensively revised phylogeny for this clade, resulting in the detection of several new lineages (fig. 2). The E-M35 polytomy reported by Trombetta et al. (2011), not fully resolved in successive studies due to the lack of informative lineages (Francalacci et al. 2013; Poznik et al. 2013; Wei et al. 2013; Lippold et al. 2014; Scozzari et al. 2014; Van Geystelen et al. 2014; van Oven et al. 2014; Hallast et al. 2015), is here completely resolved (fig. 2 and supplementary fig. S2, Supplementary Material online). Haplogroup E-M35 is bifurcated in two sister branches which are defined by Z827 and V68, respectively. Within E-Z827 we identified a new clade (E-V1515), which includes all the sub-Saharan haplogroups (E-V42, E-M293, E-V92, and E-V6) reported as E-M35 basal clades in the phylogeny by Trombetta et al. (2011) (supplementary fig. S2*A*, Supplementary Material online), as well as six new subhaplogroups. All the chromosomes previously referred to as paragroup E-M35*(×V92, V42, V6, M123, V68, M293, and V257) are now assigned to five different branches all belonging to haplogroup E-V1515 (fig. 2). This clade shows a tripartite structure, with a rare E-V1515* paragroup and two clades defined by V1486 and V1700 mutations (fig. 2). The V1486 haplogroup grouped six clades: E-M293, E-V92, and four new branches. A nested bifurcated structure is observed for the E-V1700 clade which contains the V42 branch and a sister clade (defined by V1785 and other three mutations), which joins the V6 chromosomes and 12 (former) E-M35* samples now indicated as V1785*. Haplogroup E-V1515 is mainly restricted to eastern Africa (supplementary table S7, Supplementary Material online) with the exception of the terminal clade E-M293, which has been proposed to mark an eastern to southern Africa migration (Henn et al. 2008), and V6, which we report at low frequency also in northeastern Africa.

The V68 internal phylogeny shows little but meaningful changes (fig. 2). All the V68* chromosomes in Trombetta et al. (2011) turned out to be derived at V2009 and V2955. We found six chromosomes belonging to E-V2009 haplogroup, mainly from the Mediterranean area (supplementary table S7, Supplementary Material online). Within the E-M78 subclade, we allocated most of the former E-M78* chromosomes to three new distinct branches: E-V1083*, E-V1477, and E-V259. The first is a paragroup sister to clades E-V22 and E-V13. The mutation V1477 defines a new basal branch observed only in one northern African sample (supplementary table S7, Supplementary Material online). Finally, a sister clade of E-V12, defined by V264, includes E-V65 and a new central African lineage defined by V259 (fig. 2).

### A New Deep Branch within Haplogroup E

In our sample set, we found three individuals from eastern Africa that were derived for the mutation M40, which defines haplogroup E, and ancestral for both P147 and M75 mutations, which, in turn, define the two deepest haplogroup E subclades (Karafet et al. 2008; van Oven et al. 2014). We tested these E*(xM75, P147) chromosomes for all the 13 mutations of branch 3 and for the 3 mutations of branch 4 (fig. 1, supplementary table S3 and fig. S3, Supplementary Material online). All the tested chromosomes were derived both for the mutations defining haplogroup E (branch 3) and for V3725, but ancestral for V1844 and V1865. We found that these chromosomes also share a new mutation (V44, ancestral in other haplogroup E chromosomes) that we identified by Sanger sequencing. Haplogroup E-V44 thus represents a new deep branch of haplogroup E, sister to E-P147. By this analysis, we recognized V3725 and M75 as the two mutations defining the deepest bifurcation within haplogroup E (supplementary fig. S3, Supplementary Material online).

### Phylogeography of Haplogroup E

By using both the phylogenetic information and the geographic distribution of haplogroup E clades, we performed a discrete phylogeographic analysis (Yu et al. 2010) to associate each node of the tree to one (or more) of six broad geographic regions, with an emphasis on the African continent. This analysis suggests a sub-Saharan placement for the MRCA of the haplogroup E chromosomes here analyzed (supplementary fig. S4, Supplementary Material online). Within this clade, the posterior probability (0.97) strongly favors an eastern African placement for the origin of the E-M215 diversity, as previously suggested by Semino et al. (2004) and Gebremeskel and Ibrahim (2014), whereas a northern African location is favored for the node defining the M78 subclade (posterior probability = 0.76), supporting the previous hypothesis of Cruciani et al. (2007). Despite we assigned most previous deep E-M35 eastern African clades to a single haplogroup (E-V1515), our phylogeographic analysis slightly favors an eastern African origin for E-M35 (posterior probability = 0.64). We found a new clade, defined by V1515 mutation, which originated and differentiated in eastern Africa (posterior probability = 0.99), and expanded southward in recent times as a single terminal clade (E-M293). A posterior probability of 0.92 supports a central/western African origin for haplogroup M2. Within this haplogroup, a shift in location assignment from western Africa to eastern Africa was observed along the lineage leading to a cluster of three (former) E-U209* samples, defined by V2580 mutation (supplementary fig. S4, Supplementary Material online).

## Discussion

The advent of NGS led to a huge increase in the knowledge of the worldwide human Y chromosome diversity. Two different sampling strategies were used in pioneering NGS-works: A within-population random sampling strategy (Francalacci et al. 2013; Poznik et al. 2013; Wei et al. 2013; Lippold et al. 2014; Yan et al. 2014; Hallast et al. 2015) and a sampling based on a priori knowledge about the haplogroup affiliation of the Y chromosomes to be analyzed (Scozzari et al. 2014). Although both sampling strategies have pros and cons, a lineage-based approach should enable a more accurate dissection of the phylogeny of specific haplogroups.

Here, we report an NGS-based phylogenetic refinement of human MSY haplogroup E, by disclosing the phylogenetic relationships of mutations discovered in our previous NGS-study, which used a lineage-based sampling strategy (Scozzari et al. 2014), supplemented with new data and data from Complete Genomics (Drmanac et al. 2010). The genotyping of a subset of these mutations and known additional markers (downstream to M35) in 5,222 males from different worldwide populations allowed us to define the spatial frequency distribution of the E-M35 subhaplogroup, to further refine its phylogeny, and to infer the geographic origin of its nodes.

The haplogroup E phylogeny here presented shows some relevant changes compared with the most updated and complete Y chromosome phylogenies, in which the two deepest subclades were defined by P147 and M75 mutations (Karafet et al. 2008; van Oven et al. 2014). In this study, we refine the deepest haplogroup E dichotomy, which now involves the E-M75 and the E-V3725 lineages, with the P147 mutation defining a branch sister to the newly discovered E-V44 lineage (supplementary fig. S3, Supplementary Material online). The new mutations V44 and V3725 are eligible markers to test chromosomes which resulted to belong to the paragroup E*(×M75, P147) in other studies. This paragroup, although very rare, being reported so far in two Saudi Arabian samples (Abu-Amero et al. 2009) and one southern African Bantu (Karafet et al. 2008), is of crucial relevance for phylogeographic inferences about the origin of haplogroup E and linked hypotheses about movements out of and back to Africa (Hammer et al.1998; Underhill and Kivisild 2007; Scozzari et al. 2014). Keeping these caveats in mind, our phylogeographic analysis, based on thousands of samples worldwide, suggests that the radiation of haplogroup E started about 58 ka, somewhere in sub-Saharan Africa, with a higher posterior probability (0.73) for an eastern African origin. Moreover, it seems that the next five major dichotomies also occurred in eastern Africa (posterior probabilities ranging 0.84–0.97) in a time frame of about 15 ky (55–40 ka), underlying the importance of this region for the early differentiation of this widespread haplogroup and for the peopling of the entire continent (supplementary fig. S4, Supplementary Material online).

**Fig. 3.**
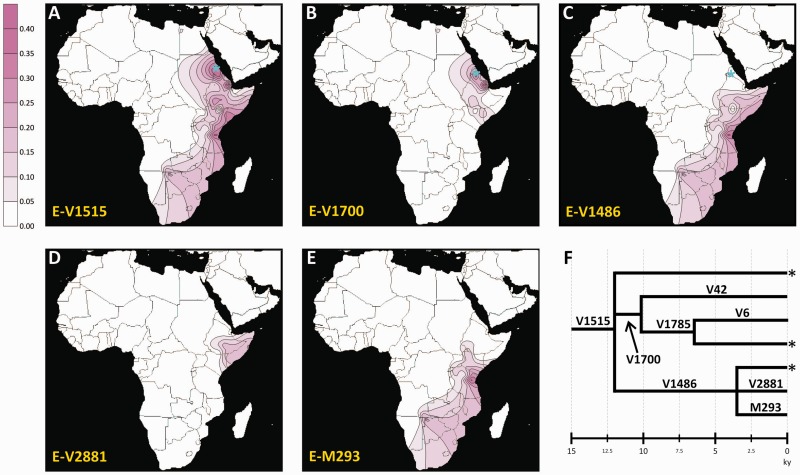
—Maps of the observed frequencies for haplogroup E-V1515 and its major subhaplogroups. (*A*) Haplogroup E-V1515, (*B*) haplogroup E-V1700, (*C*) haplogroup E-V1486, (*D*) haplogroup E-V2881, (*E*) haplogroup E-M293, (*F*) simplified phylogeny of E-V1515, showing the estimated age of the nodes (ky). Place of sampling of chromosomes carrying rare paragroups E-V1515*, E-V1785*, and E-V1486* is shown as blue asterisks in panels (*A*), (*B*), and (*C*), respectively. The sampling locations for the populations used in this analysis are shown in supplementary figure S5, Supplementary Material online.

One of the most interesting findings of our phylogeographic refinement is the identification of a new clade (E-V1515), which originated about 12 ka (95% CI: 8.6–16.4) in eastern Africa (posterior probability = 0.99) where it is currently mainly distributed. This clade includes all the sub-Saharan chromosomes belonging to the former paragroup E-M35*(×V92, V42, V6, M123, V68, M293, and V257), as well as all the sub-Saharan haplogroups (E-V42, E-M293, E-V92, and E-V6) reported as E-M35 basal clades in a previous phylogeny (Trombetta et al. 2011) (fig. 2). We observed the highest frequency and diversity of this haplogroup in the northern part of the Horn of Africa (present day Eritrea and northern Ethiopia), where the majority of the deepest E-V1515 subhaplogroups and paragroups were found (fig. 3 and supplementary table S7, Supplementary Material online). In the southern part of the Horn (southern Ethiopia, Somalia and northern Kenya), haplogroup E-V1515 is almost exclusively represented by the recent (3.5 ka; 95% CI: 1.7–5.9 ka) subhaplogroup E-V1486. Further south, in southern Kenya and southern Africa, a single E-V1486 terminal clade, known as E-M293 (Henn et al. 2008), was found (fig. 3). This phylogeographic pattern is strongly suggestive of human movements from the northern part of the Horn to the Ethiopian/Kenyan borders between 12 ka (the coalescence of E-V1515) and 3.5 ka (the coalescence of E-V1486), and from here toward southern Africa across the equatorial belt in more recent times (supplementary fig. S4, Supplementary Material online). Haplogroup E-M293 has been previously hypothesized to mark a recent gene flow (about 2 ka) through Tanzania to southern Africa, as a consequence of a migration of non-Bantu speaker pastoralists (Henn et al. 2008). Our data on the distribution and phylogeny of the E-V1515 haplogroup support and extend this hypothesis. We propose that the migration marked by the E-M293 haplogroup could be the final step of a north-to-south range expansion linked to different branches of E-V1515, which initially involved people from Eritrea (and possibly northern Sudan, not sampled here). This migratory route is concordant in time and space with archeological evidence for early domestication of African cattle in northeastern Africa about 10 ka, southward climate-driven movements of herders into southern Ethiopian highlands and Turkana basin (northern Kenya) around 4 ka, and a subsequent subequatorial pastoralist expansion toward southern Kenya/Tanzania and southern Africa not before 3 ka (Ehret 2002; Marshall and Hildebrand 2002; Lesur et al. 2014; Wright 2014). Within haplogroup E-V1515, we also remarked a striking parallelism in the geographic distribution of the MSY sister clades E-V1486 and E-V1700 (fig. 3) with respect to mutations C-14010 and G-13907 found within the enhancer of the autosomal *LCT* gene (Tishkoff et al. 2007; fig. 3 in Ranciaro et al. 2014). Both C-14010 and G-13907 have been associated with lactase persistence in adulthood and, similarly to MSY mutations V1486 and V1700, are essentially eastern African specific, recent, and found at high frequencies among pastoralist groups (Ingram et al. 2007, 2009; Tishkoff et al. 2007; Jones et al. 2013; Ranciaro et al. 2014). Similarly to C-14010, E-V1486 is found at high frequencies in pastoralist populations from Kenya and Tanzania, (and further south at lower frequencies), whereas E-V1700 mirrors the distribution of the lactase mutation G-13907, being observed at its highest frequency among Cushitic populations from the Horn. Considering the spatial–temporal congruencies with other pieces of evidence coming from archaeology as well as genetics, we propose that the observed phylogeographic trajectories of haplogroup E-V1515 could be the consequence of multistep southward movements of early pastoralists from a northeastern African motherland across the equatorial belt.

For more than 20 years, the MSY diversity has been used by geneticists to solve specific forensic cases in which routinely used autosomal markers could be uninformative. These cases range from fatherless paternity tests to generation of male-specific profiles in presence of strongly unbalanced male–female mixtures (Roewer et al. 2009; Maiquilla et al. 2011). To date, these analyses were mainly based on highly variable microsatellite markers (Y-STR), but, with the introduction of NGS technologies, the forensic interest on Y-SNPs has rapidly increased (Xue and Tyler-Smith 2010). One of the most important advantages in the forensic use of Y-SNPs with respect to Y-STRs is their potential as ancestry informative markers (AIMs) in suspect-less cases, allowing hypotheses to be formulated on the geographic origin/ethnicity of a DNA trace (Wetton et al. 2005; van Oven et al. 2013; Ralf et al. 2014), with the due cautions (King et al. 2007). To be considered as a useful AIM, a de novo identified Y-SNP/haplogroup should be restricted to a certain geographic area and its frequency distribution must be known in a worldwide population sample. In this study, we report the identification of several new geographically restricted haplogroups and their frequency distribution in a large sample of 118 worldwide populations. Examples of new Y-AIMs include V42, V1785, and V2881 (limited to and highly prevalent in the Horn of Africa); V259 found only in central Africa (supplementary table S7, Supplementary Material online); and V2580, which seems to identify a southeastern African-specific branch of E-M2 (Cruciani F, Trombetta B, unpublished data). These and other SNPs from our survey can improve the ancestry detection performance of high resolution multiplex tools such as the NGS-based genotyping method designed by Ralf et al. (2014).

In conclusion, we show that haplogroup E and, more specifically, its subhaplogroup E-M35, previously refractory to several subsequent phylogenetic refinements, contain a lot of phylogenetic and phylogeographic information which can be useful for evolutionary and forensics purposes. These results emphasize the relevance of analyzing large population samples to fully disclose the phylogenetic information hidden in the great number of mutations that have been reported in recent Y chromosome NGS studies. In general, we look forward to large population studies that make use of newly available mutation resources as a promising way to reveal the paternal side of our evolutionary history, like it has been already done in a very limited number of studies so far (Rootsi et al. 2013; Underhill et al. 2015).

## Supplementary Material

Supplementary figures S1–S5 and tables S1–S7 are available at *Genome Biology and Evolution* online (http://www.gbe.oxfordjournals.org/).

Supplementary Data
