# Supplementary material for: Phylogeographic Refinement and Large Scale Genotyping of Human Y Chromosome Haplogroup E Provide New Insights into the Dispersal of Early Pastoralists in the African Continent
Source: Genome Biol Evol. 2015 Jun 24;7(7):1940–50. doi: 10.1093/gbe/evv118 (PMC4524485; doi:10.1093/gbe/evv118)
Supplement: Supplementary Data [file supp_7_7_1940__index.html]

Phylogeographic Refinement and Large Scale Genotyping of Human Y Chromosome Haplogroup E Provide New Insights into the Dispersal of Early Pastoralists in the African Continent — Supplementary Data 

# Phylogeographic Refinement and Large Scale Genotyping of Human Y Chromosome Haplogroup E Provide New Insights into the Dispersal of Early Pastoralists in the African Continent

## Supplementary Data

files

- Supplementary Data - pdf file
- Supplementary Data - xlsx file
