# Supplementary material for: Phylogeographic Refinement and Large Scale Genotyping of Human Y Chromosome Haplogroup E Provide New Insights into the Dispersal of Early Pastoralists in the African Continent
Source: Genome Biol Evol. 2015 Jun 24;7(7):1940–50. doi: 10.1093/gbe/evv118 (PMC4524485; doi:10.1093/gbe/evv118)
Supplement: Supplementary Data [file supp_evv118_Supplementary_figures_1-5_revised.pdf]

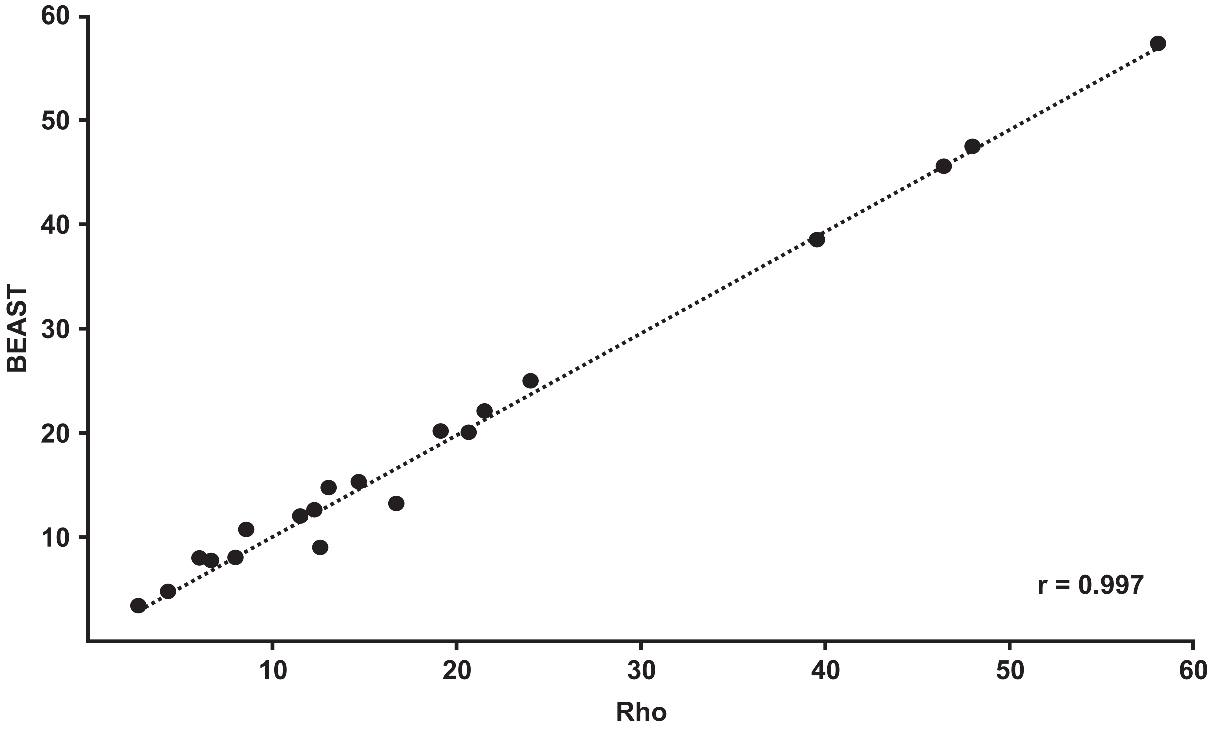

Figure S1: Scatter plot of ages (kya) for the nodes of the tree shown in fig. 1. X-axis: rho estimates; Y-axis: BEAST estimates.

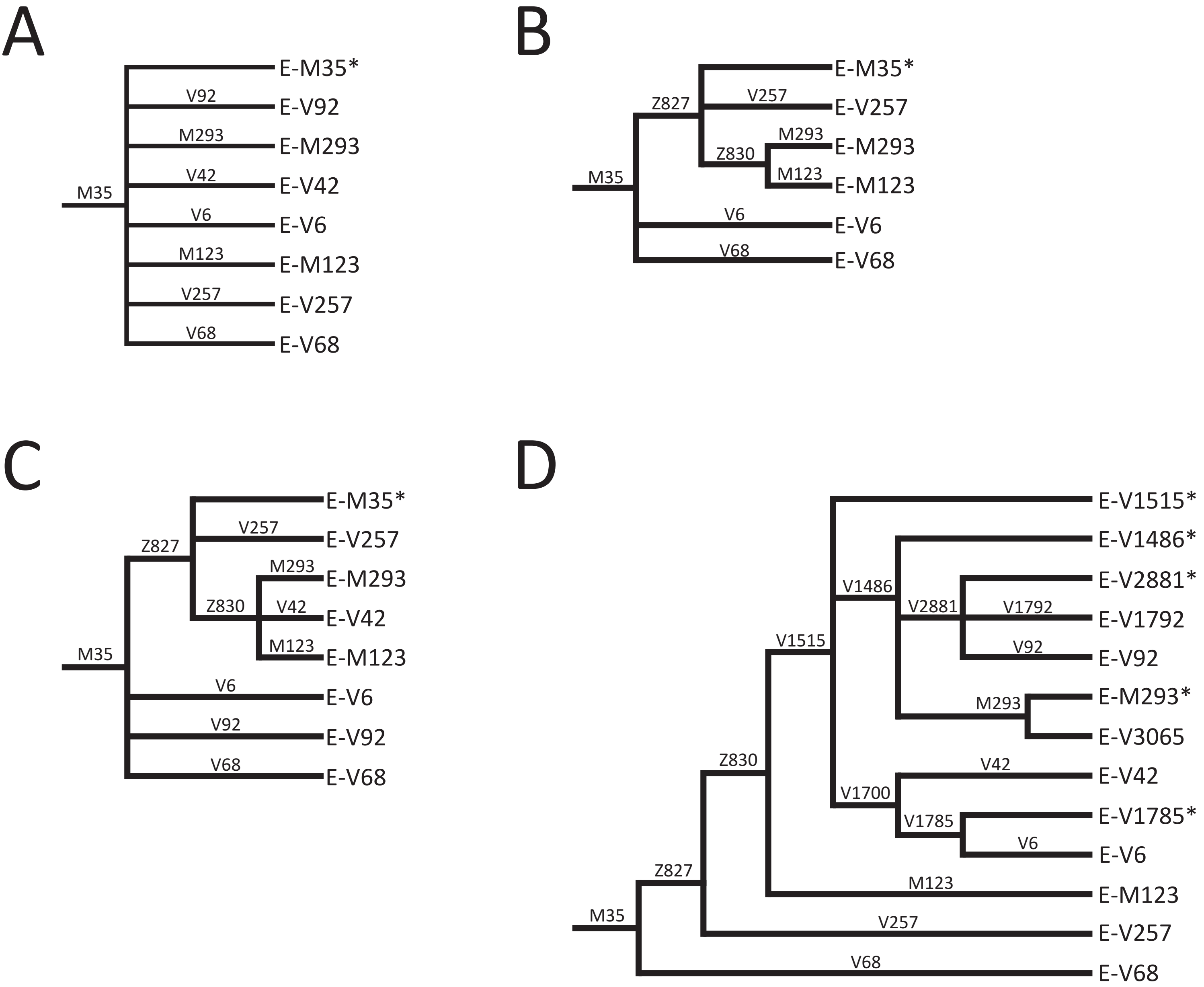

Figure S2: Phylogenetic relationships among the major basal clades of the E-M35 haplogroup as reported in: A) Trombetta et al. 2011; B) van Oven et al. 2014; C) ISOGG website (http://www.isogg.org/tree/index.html, version 10.14 – 10/2/2015); D) present study.

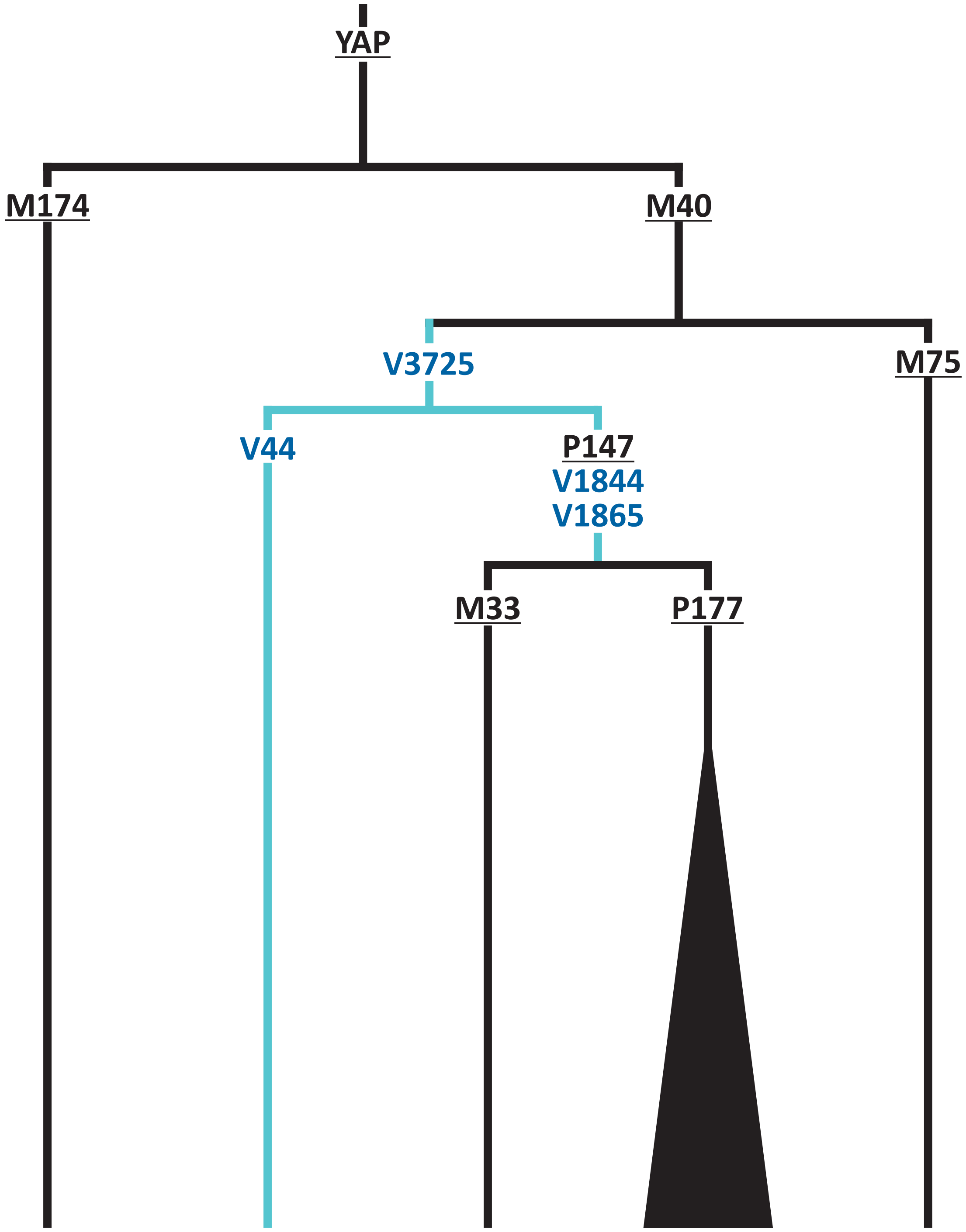

<table>
<tr><td rowspan="1">Former nomenclature (by SNP)</td><td>D-M174</td><td>E-M40*</td><td>E-M33</td><td>E-P177</td><td>E-M75</td></tr>
<tr><td rowspan="2">New nomenclature (by SNP)</td><td rowspan="2">D-M174</td><td>E-V44</td><td>E-M33</td><td>E-P177</td><td rowspan="2">E-M75</td></tr>
<tr><td colspan="3">E-V3725</td></tr>
</table>

Figure S3: Phylogenetic refinement of the deepest clades of haplogroup E. In blue: new mutations and branches. At the bottom, former and new nomenclature for each branch is shown. The 13 mutations (branch 3 of figure 1) phylogenetically equivalent to M40 are not here represented.

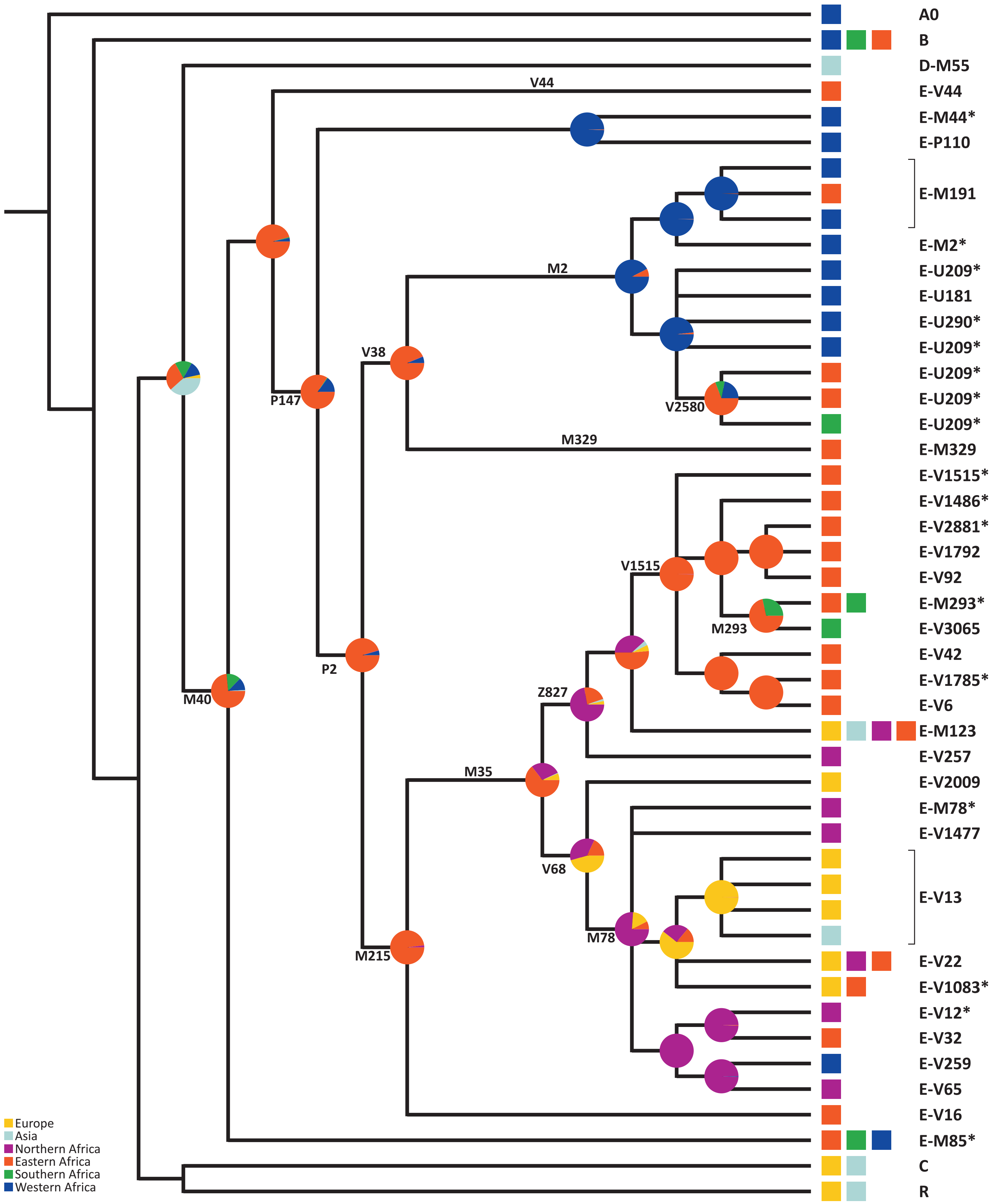

Figure S4: Results of the phylogeographic analysis using a Bayesian approach (see text).
The tree shows the phylogenetic relationships among all the "sequenced" and the "genotyped" samples belonging to haplogroup DE, with the addition of haplogroups A, B, C and R. Pie charts reflects the posterior probability of ancestral geographic location for each node, as coded in the legend. The name and the geographic distribution for each terminal branch of the tree are reported to the right.

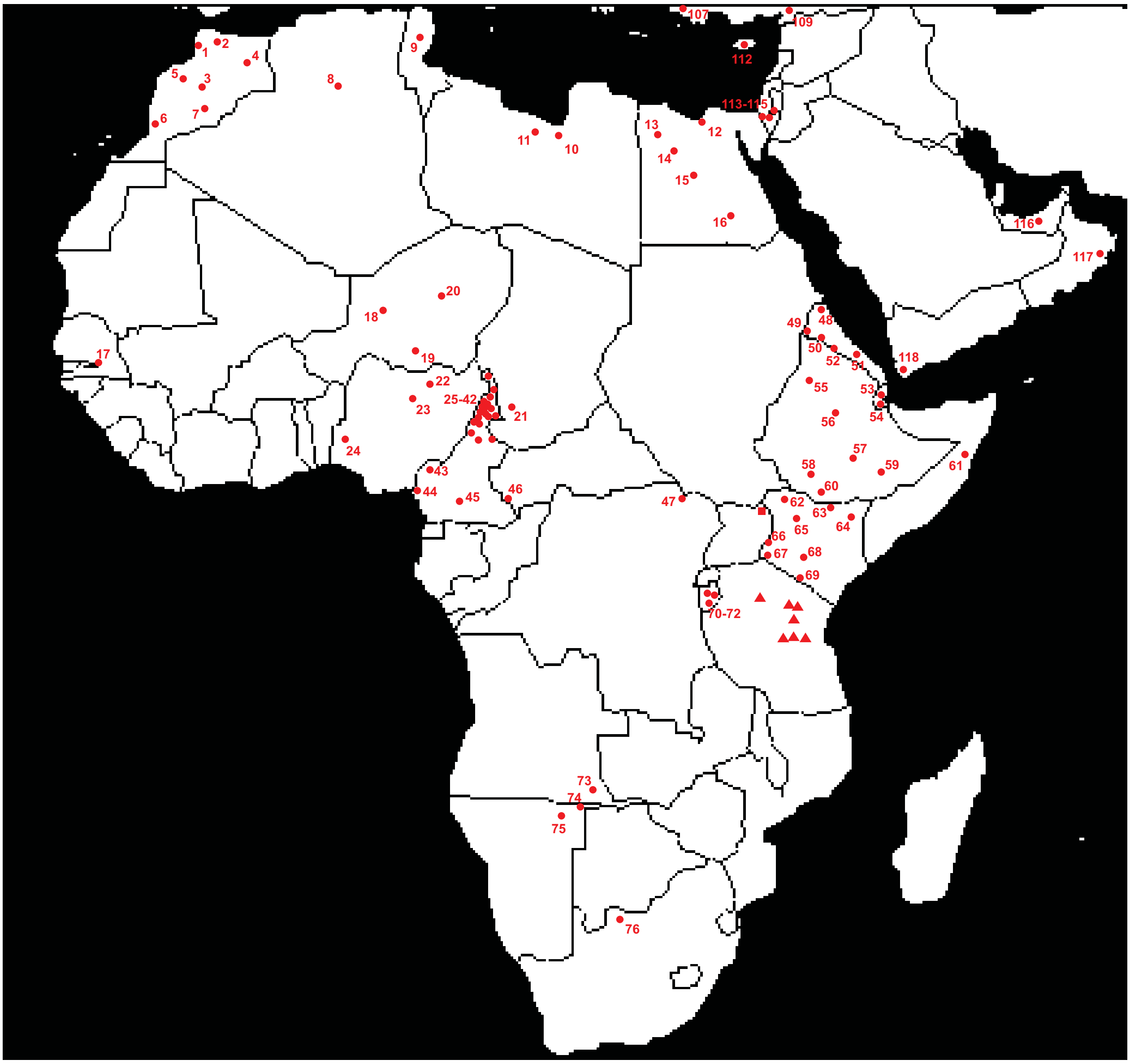

Figure S5: Location of populations used for the frequency maps. Circles: data from the present study (population numbers as reported in supplementary table S6); squares: data from Gomes et al. (2010); triangles: data from Henn et al. (2008).
